# Resting heart rate variability as a predictor of exercise response in mild post-COVID: insights from a six-minute step test

**DOI:** 10.1186/s12938-025-01499-x

**Published:** 2025-12-14

**Authors:** Aldair Darlan Santos-de-Araújo, Daniela Bassi-Dibai, Nelson Francisco Serrão Júnior, Bárbara Rocha de Oliveira Garcia, Renan Shida Marinho, Paula Angélica Ricci, Shane A. Phillips, Audrey Borghi-Silva

**Affiliations:** 1 Department of Physical Therapy, Cardiopulmonary Physical Therapy Laboratory, Federal University of São Carlos, Rodovia Washington Luiz, São Carlos, SP 13565-905 Brazil; 2 Department of Dentistry, CEUMA University, São Luís, MA Brazil; 3 Postgraduate Program in Management in Health Programs and Services, CEUMA University, São Luís, MA Brazil; 4 State University of Maranhão, Itapecuru-Mirim, MA Brazil; 5 Physical Therapy Department, Federal University of Pampa, Uruguaiana, RS Brazil; 6 Physical Therapy Department, Central Paulista University Center (UNICEP), São Carlos, SP Brazil; 7 Postgraduate Program Inter-Units of Bioengineering, University of São Paulo, São Carlos, SP Brazil; 8 Department of Physical Therapy, College of Applied Health Sciences, University of Illinois Chicago, Chicago, IL USA

**Keywords:** Heart rate variability, Functional capacity, Six-minute step test, COVID-19

## Abstract

**Background:**

COVID-19 may impair autonomic and cardiorespiratory function, even in mild cases, resulting in reduced heart rate variability (HRV) and diminished functional capacity. Given their shared regulatory pathways, resting HRV may serve as a non-invasive predictor of oxygen uptake (V̇O_2_) during the six-minute step test (6MST).

**Objective:**

To investigate whether resting short-term HRV can predict V̇O_2_ during the 6MST in individuals recovering from post-COVID.

**Methods:**

In this cross-sectional study, adults recovering from mild COVID underwent assessment of autonomic modulation via short-term HRV and cardiorespiratory response during 6MST. HRV was recorded under standardized resting conditions. Gas exchange was measured throughout the 6MST. Spearman correlation and multiple linear regression analyses were performed to test associations between HRV parameters and V̇O₂ expressed in milliliters per kilogram per minute (mL·kg^−1^·min^−1^).

**Results:**

Data from 45 participants were analyzed. Several HRV variables demonstrated statistically significant correlations with V̇O₂ and were therefore included in the simple linear regression analysis: SDNN (ms) (rho = 0.587), RMSSD (ms) (rho = 0.430), RR Tri (rho = 0.594), TINN (rho = 0.596), SD1 (ms) (rho = 0.431), SD2 (ms) (rho = 0.609), ApEn (rho = − 0.388), and DFA α2 (rho = − 0.404). Multiple linear regression showed that SD2 (ms) and sex were significant predictors of V̇O₂ (mL·kg^−1^·min^−1^) at the peak of the 6MST, while weight (kg) and age (years) were not. The model explained 50.7% of the variance (adjusted R^2^ = 0.507, *p* < 0.001).

**Conclusion:**

Several HRV parameters were significantly correlated with V̇O₂, indicating associations between cardiac autonomic modulation and aerobic performance. Among these, SD2 together with sex, emerged as significant predictors of VO₂ at the peak of the 6MST. Future studies will be needed to combine HRV indices with clinical outcomes in order to determine the mechanisms of V̇O₂ variability in post-COVID populations.

## Introduction

COVID-19 infection affects the respiratory, cardiovascular, musculoskeletal, and nervous systems, resulting in a multifaceted clinical presentation and long-lasting sequelae, regardless of disease severity [1–3]. One of the primary mechanisms proposed to explain the extent of these effects is the neurotropism of SARS-CoV-2 [4]. The virus’s ability to impact the central nervous system directly contributes to systemic dysregulation, impairing autonomic function integrity and disrupting the modulation of cardiovascular and respiratory responses [4].

Individuals recovering from COVID-19 frequently exhibit impaired cardiac autonomic modulation, as evidenced by reduced heart rate variability (HRV), a marker of autonomic nervous system function, regardless of infection duration, clinical severity, or hospitalization status [5–7]. These autonomic alterations have been observed even in mild or asymptomatic cases, often within weeks after diagnosis [5–9]. Briefly, HRV reflects the balance between sympathetic and parasympathetic activity, with lower values indicating reduced vagal tone and autonomic dysfunction [10–12]. Clinically, decreased HRV has been associated with increased cardiovascular risk, poorer prognosis, and a diminished ability to adapt to physiological stressors [10]. Similarly, individuals recovering from COVID-19 have consistently demonstrated declines in functional capacity and exercise performance, as evidenced by both field-based and laboratory assessments [13–16].

Together, these findings suggest that autonomic dysfunction and cardiorespiratory limitations may coexist, given their shared mechanisms related to stress response and cardiovascular control [17]. Among the tools used to assess functional capacity, the six-minute step test (6MST) is a valid, reproducible, and reliable option. While typically considered submaximal, the test can elicit maximal effort depending on individual physiological status. This occurs through recruitment of large muscle groups and the consequent rise in oxygen uptake (V̇O_2_), alongside increased chronotropic and inotropic responses. These demands may also lead to shifts in energy substrate utilization, potential lactate accumulation, and/or impaired oxygen delivery to muscles, reduced efficiency in oxygen transport to the muscles, factors potentially exacerbated by COVID-19-induced vascular dysfunction [15, 18, 19].

Resting HRV may serve as a physiological predictor of oxygen consumption during exercise due to its direct relationship with cardiac autonomic regulation. Specifically, HRV measures that reflect parasympathetic activity are closely associated with vagal tone, which influences heart rate, stroke volume, and overall cardiac efficiency [10, 11, 20–23]. Individuals with higher resting vagal modulation typically demonstrate more efficient oxygen delivery and utilization during exercise [24–26]. This autonomic adaptability enhances both perfusion and muscle oxygen extraction, key determinants of oxygen consumption [27].

Despite the growing interest in understanding post-COVID-19 sequelae, little is known about the physiological mechanisms that link autonomic modulation and cardiorespiratory responses during functional exercise tests. Resting HRV has been widely investigated as a non-invasive marker of autonomic regulation and has shown associations with aerobic fitness and exercise tolerance in different populations [10, 20]. However, its potential role as a predictor of V̇O₂ during submaximal tests, such as the 6MST, remains largely unexplored, particularly in individuals recovering from mild COVID-19. Although the 6MST is considered a submaximal exercise test, inherent contraindications make it difficult to estimate V̇O₂ and functional capacity in all individuals [28]. Therefore, alternative non-invasive tools, such as resting HRV, may provide valuable information to predict exercise responses and better characterize functional capacity in this population. Given the shared regulatory basis of autonomic and cardiorespiratory systems, we hypothesize that resting HRV may influence and even predict oxygen uptake during the 6MST. Therefore, the aim of this study was to explore the predictive capacity of resting HRV to estimate V̇O_2_ during the 6MST in individuals recovering from mild COVID-19, assessed within the acute recovery phase.

## Results

Initially, 46 volunteers were enrolled in the study. One subject terminated the 6MST before completing the first minute and did not resume performance during the remaining time, leaving a final sample of 45 volunteers for analysis.

### Clinical, sociodemographic, and anthropometric characteristics

The mean age was 34 ± 11 years, and 60% were women. The mean BMI was 27.09 ± 5.89 kg/m^2^. Pulmonary function testing showed a mean FEV₁ of 3.28 ± 0.67 (L) (94.91 ± 12.05% of predicted) and a mean FVC of 3.99 ± 0.84 (L) (97.67 ± 11.68% of predicted), with an FEV₁/FVC ratio of 82.32 ± 6.44%. These values are consistent with normal pulmonary function, as the FEV₁, FVC, and FEV₁/FVC ratio were within expected reference ranges, indicating neither a restrictive nor obstructive pattern. A more detailed description of physical activity levels, risk factors, comorbidities, and symptomatology can be seen in Table 1.

**Table 1:** Clinical and anthropometric characteristics of participants included in the analyses

Variables	All included (*n* = 45)
Age (years)	34 ± 11
Gender	
Male	18 (40)
Female	27 (60)
Height (m)	1.69 ± 0.10
BMI (kg/m^2^)	27.09 ± 5.89
Pulmonary function	
Spirometry	
FEV_1_ (L)	3.28 ± 0.67
FEV_1_ (%, predicted)	94.91 ± 12.05
FVC (L)	3.99 ± 0.84
FVC (%, predicted)	97.67 ± 11.68
FEV_1_/FVC	82.32 ± 6.44
Risk factors	
Asthma	1 (2)
Systemic arterial hypertension	8 (18)
Obesity	8 (18)
Thyroid dysfunction	2 (4)
Dyslipidemia	3 (7)
Diabetes mellitus	2 (4)
Depression	7 (16)
Former smoking	1 (2)
Symptomatology	
Fever	34 (76)
Cough	30 (67)
Sore throat	26 (58)
Breathlessness	13 (29)
Diarrhea	9 (20)
Nausea	5 (11)
Vomiting	4 (9)
Headache	22 (49)
Runny nose	21 (47)
Asthenia	20 (44)
Chills	13 (29)
Nasal congestion	24 (53)
Anosmia	6 (13)
Ageusia	5 (11)

kg, kilos; m, meter; BMI, body mass index; Kcal, kilocalories; %, percentage; mMRC, Modified Medical Research Council; FVC, forced vital capacity; L, liter; %, percentage; FEV_1_, forced expiratory volume in first second; FEV_1_/FVC, ratio between forced vital capacity and forced expiratory volume in first second;

### Resting HRV and correlation with V̇O_2_ (mL·kg^−1^·min^−1^)

Data on resting HRV and Spearman’s correlations with V̇O_2_ (mL·kg^−1^·min^−1^) can be seen in Table 2. We found moderate positive and significant correlations between V̇O_2_ (mL·kg^−1^·min^−1^) and the SDNN (ms), RMSSD (ms), RR Tri, TINN, SD1 (ms), and SD2 (ms). A weak negative correlation was found between V̇O_2_ (mL·kg^−1^·min^−1^) and the ApEn variable, while moderate negative correlations were observed in the DFA α2 variable (*p* < 0.05).

**Table 2:** Resting HRV parameters in post‑COVID subjects and their correlation with oxygen uptake during 6MST

HRV parameters	Mean ± SD or median (IQR25–75)	Spearman’s correlation V̇O_2_ (mL·kg^−1^·min^−1^)	*P* value
Time domain			
Mean RR (ms)	768.80 (714.70–873.70)	0.248	0.100
SDNN (ms)	26.87 (18.77–40.41)	0.587	< 0.0001^*^
MeanHR (bpm)	75.72 ± 11.84	− 0.250	0.098
RMSSD (ms)	24.78 (18.60–34.21)	0.430	0.003^*^
RRTri	7.46 (4.88–11.33)	0.594	< 0.0001^*^
TINN	147.00 (97.00–195.00)	0.596	< 0.0001^*^
Frequency domain			
LF (n.u.)	53.87 ± 19.87	− 0.045	0.769
HF (n.u.)	46.06 ± 19.87	0.047	0.761
LF/HF	1.16 (0.63–2.07)	− 0.050	0.744
Nonlinear analysis			
SD1 (ms)	17.54 (13.17–24.23)	0.431	0.003^*^
SD2 (ms)	33.05 (22.50–51.34)	0.609	< 0.0001*
SD2/SD1	1.96 ± 0.60	0.090	0.557
Apen	1.12 ± 0.11	− 0.388	0.008^*^
SampEn	1.64 ± 0.30	− 0.255	0.092
DFA α1	1.03 ± 0.30	0.021	0.892
DFA α2	0.35 ± 0.16	− 0.404	0.006^*^

6MST, six-minute step test; SD, standard deviation; IQR, interquartile range; HRV, heart rate variability; SNS, sympathetic nervous system; PSN, parasympathetic nervous system; Mean RR, average R–R interval duration; ms, milliseconds; SDNN, standard deviation of NN intervals; Mean HR, mean heart rate; bpm, beats per minute; RMSSD, square root of successive mean squared differences of R-R; RR Tri, integral of the density of the RR interval histogram divided by its height; TINN, baseline width of the RR interval histogram; LF, low-frequency; HF, high-frequency; n.u., normalized units; SD1, Poincaré plot standard deviation perpendicular the line of identity; SD2, Poincaré plot standard deviation along the line of identity; ApEn, approximate entropy, which measures the regularity and complexity of a time series; SampEn, Sample entropy, which measures the regularity and complexity of a time series; DFA α1, detrended fluctuation analysis, which describes short-term fluctuations; DFA α2, detrended fluctuation analysis, which describes long-term fluctuations

^*^Statistical significance for Spearman (*ρ*) correlation (*p* < 0.05)

### 6MST performance, cardiorespiratory, and hemodynamics responses

The participants’ functional capacity, assessed by the 6MST, showed a mean of 145 ± 31 steps, corresponding to 82% ± 14% of the predicted value according to Albuquerque. Regarding cardiorespiratory responses, the mean V̇E was 49.79 ± 17.51 L/min, with a RER of 0.95 ± 0.10. The RR reached 32.69 ± 7.29 breaths per minute. V̇O₂ was 1548.68 ± 486.40 mL/min, while the median and interquartile range (25^th^–75th) of V̇CO₂ was 1511 (1056–1851) mL/min. Following body weight adjustment, V̇O₂ the median was 18.90 (15.85–26.02) mL·kg^−1^·min^−1^. A detailed description of the performance, cardiovascular, and cardiorespiratory responses can be found in Table 3.

**Table 3:** Functional capacity, hemodynamic and cardiorespiratory responses in the 6MST

Variables	Mean ± SD or median (IQR25–75)
Functional capacity and hemodynamic outcomes	
Steps from 6MST	145 ± 31
%Predicted by Albuquerque	82 ± 14
HR (bpm) rest	84 (52–91)
HR (bpm) peak	156 ± 21
HR (bpm) rec 1′	124 ± 23
HR (bpm) rec 3′	106 ± 20
HR (bpm) rec 6′	98 ± 17
SBP (mmHg) rest	112 (110–128)
DBP (mmHg) rest	78 (72–82)
SBP (mmHg) peak	150 (135–170)
DBP (mmHg) peak	80 (74–84)
SBP (mmHg) rec 6′	118 (110–124)
DBP (mmHg) rec 6′	76 (72–80)
SpO_2_ (%) rest	97 ± 3
SpO_2_ (%) peak	96 ± 3
SpO_2_ (%) rec 6′	98 ± 2
BORG dyspnea peak	3 (1–3)
BORG fatigue lower limbs peak	3 (1–5)
Cardiorespiratory responses	
V̇_E_ (L/min)	49.79 ± 17.51
RER	0.95 ± 0.10
RR (minute)	32.69 ± 7.29
V̇O_2_ (mL·min)	1511 (1056–1851)
V̇CO_2_ (mL· min)	1478.24 ± 513.11
V̇O_2_ (mL·kg^−1^·min^−1^)	18.90 (15.85–26.02)

Values are mean ± standard deviation or median and interquartile range (IQR, 25th–75th percentile)

6MST, six-minute step test; %: percentage; HR, heart rate; bpm, beats per minute; rec, recovery; ‘, minute; SBP, systolic blood pressure; mmHg, millimeters of mercury; DBP, diastolic blood pressure; SpO_2_, peripheral oxygen saturation; V̇_E_, minute ventilation; L, liters; min, minute; RR, respiratory rate; V̇O_2_, oxygen uptake; RER, respiratory exchange ratio; ml: milliliter; V̇CO_2_, carbon dioxide production; kg, kilos

### Regression analyses and the predictive capacity of HRV variable to estimate V̇O_2_ (mL·kg^−1^·min^−1^)

A multiple linear regression (Table 4) was performed to examine the influence of HRV indices on V̇O₂ (mL·kg^−1^·min^−1^) at the peak of the 6MST. The model was statistically significant (ANOVA, *p* < 0.001) and explained 50.7% of the variance in V̇O₂ (Adjusted R^2^ = 0.507). Among the predictors, SD2 (ms) (*β* = 0.087, *p* = 0.015) and sex (0 = female, 1 = male; *β* = 7.071, *p* < 0.001) contributed significantly to the model, whereas weight (kg) (*β* = − 0.039, *p* = 0.384) and age (ms) (*β* = − 0.090, *p* = 0.228) did not. Collinearity diagnostics indicated no multicollinearity issues (tolerance = 0.682–0.910; VIF = 1.098–1.466). Residuals were independent (Durbin–Watson = 2.002) and normally distributed (Shapiro–Wilk = 0.307).

**Table 4:** Multiple linear regression to determine the influence of clinical variables in the V̇O_2_ (mL·kg^−1^·min^−1^)

Model	Non-standard coefficients		Standardized coefficients	*t*	*P* value	Collinearity statistics	
	*β*	Erro	*β*			Tolerance	VIF
Constant	20.076	4.993		4.021	< 0.0001^*^		
SD2 (ms)	0.087	0.034	0.345	2.567	0.015^*^	0.682	1.466
Sex (0, female; 1, male)	7.071	1.469	0.560	4.815	< 0.0001^*^	0.910	1.098
Weight (kg)	− 0.039	0.044	− 0.103	− 0.882	0.384	0.900	1.111
Age (years)	− 0.090	0.074	− 0.160	− 1.225	0.228	0.720	1.390
Dependent variable: V̇O_2_ (mL·kg^−1^·min^−1^)							
R = 0.746							
R^2^ = 0.556							
Adjusted R^2^ = 0.507							
ANOVA p value ≤ 0.001^*^							
Durbin–Watson = 2.002							
Standardized residual Shapiro–Wilk = 0.307							

VIF: variance inflation factor; SD2: Poincaré plot standard deviation along the line of identity; ms: milliseconds; kg: kilos

^*^Statistical significance (*p* < 0.05)

## Discussion

This study aimed to explore the association between short-term resting HRV and V̇O₂ (mL·kg^−1^·min^−1^) measured during the 6MST. Our exploratory analysis revealed that several HRV indices were significantly associated with V̇O₂ when assessed using correlation analysis, particularly SD2, which showed the strongest correlation with V̇O₂. When controlling for other confounding variables (age, sex, and weight) that are classically recognized for their influence on oxygen consumption, the multiple linear regression model maintained its association in predicting V̇O₂ alongside the other variables in the model. These findings support the potential of resting HRV as a non-invasive indicator of submaximal cardiorespiratory performance.

From a physiological perspective, and in line with our findings, it is plausible that both parasympathetic and sympathetic components of HRV are associated with oxygen consumption during the peak of the 6MST. These two autonomic branches play distinct but complementary roles in cardiovascular regulation and contribute to autonomic control during physical exertion. Resting HRV reflects the dynamic balance between parasympathetic and sympathetic activity, which governs heart rate modulation and systemic hemodynamics. Higher vagal modulation—typically represented by increased values of RMSSD (ms), and SD1 (ms)—suggests efficient parasympathetic regulation and has been associated with better cardiovascular performance and oxygen efficiency [29, 30]. Conversely, elevated sympathetic activity may indicate increased physiological arousal or reduced autonomic flexibility, which, when present at rest, may impair exercise capacity [30]. In addition, global HRV measures such as SDNN (ms), TINN (ms), RR Tri, SD2 (ms), and ApEn offer broader insight into overall autonomic variability and system complexity [10, 31].

The predictive potential of HRV for V̇O₂ has been explored previously [32–38]. Although the results appear promising, they still raise some perplexity due to the discrepancies observed among the findings. Moreover, despite being a methodology investigated over a considerable period, the available evidence remains limited, revealing a relatively elementary stage in understanding the association between resting HRV and cardiorespiratory fitness, even considering that the autonomic nervous system plays a central role in regulating cardiovascular function and adapting to physical exertion.

A recent study using mediation analysis in individuals with post-COVID-19 syndrome suggested that maximal oxygen uptake (VO₂max), assessed via cardiopulmonary exercise test, may be a contributing factor in the autonomic dysfunction observed in this population [30]. Patients with COVID-19 exhibited impaired autonomic modulation at rest, as evidenced by short-term HRV metrics recorded with a portable heart rate monitor compared to healthy patients. The results revealed a partial mediating effect of VO₂max on the relationship with RMSSD (ms) and SDNN (ms), suggesting that an interaction between cardiorespiratory fitness and autonomic function may exist [30].

These findings underscore the central role of the autonomic nervous system in limiting functional capacity, potentially through mechanisms such as cerebral hypoperfusion, neuroinflammation, or direct neuroinvasion of SARS-CoV-2 in critical brain regions, including the hypothalamus [4, 30]. While offering important insights into the pathophysiology of long COVID, the authors acknowledged some methodological limitations, including the cross-sectional design, small sample size, and the need for future longitudinal studies to validate and expand upon these findings [30].

When investigating the predictive capacity of 24-h HRV on exercise capacity in young patients with Cardiac Syndrome X, it was observed that frequency-domain indices, including very low frequency, low frequency, and total power (ms^2^), were independently associated with the ability to reach 90% of the age-predicted maximum heart rate during a treadmill exercise test [33]. In contrast, the high-frequency component of HRV did not reach statistical significance, suggesting that parameters related to sympathetic modulation and overall autonomic variability may play a more relevant role in predicting chronotropic response to exercise in this population [33].

The V̇O₂ assessed through a maximal exercise test using a treadmill protocol in overweight or obese children showed that although resting HRV parameters [RMSSD (ms), pNN50 (%), SDNN (ms), total power (ms)^2^, HF (ms^2^), LF (ms^2^), and LF/HF] had a predictive capacity for the peak oxygen uptake obtained during the test when reported as standardized parameters. This observation became non-significant when the dependency of HRV on heart rate is considered, that is, when HRV variables are corrected by heart rate [38]. Additionally, the associations between HRV and cardiorespiratory fitness were not significant when potential confounding factors were controlled (sex, maturation, adiposity, moderate-to-vigorous physical activity, energy intake, circadian-related variables, and heart rate), leading to the conclusion that HRV parameters do not provide additional information about cardiorespiratory fitness in this population [38].

We must acknowledge that, although the study was methodologically well-designed and appropriate statistical methods were employed to answer the authors’ proposed objectives, the multiple regression models used did not test for diagnostic criteria of collinearity between the independent variables included in the models. Therefore, it remains unclear to what extent these results may have been influenced by the lack of such testing, particularly given that sex and body fat percentage were included in the model, and these variables may exhibit multicollinearity, potentially distorting the true relationships between the predictors and the outcome. Including variables like sex and body fat percentage, which are highly correlated, without appropriate statistical controls—such as assessing collinearity before performing multiple regression, can lead to inflated standard errors or biased estimates. This, in turn, limits the ability to determine the independent effects of each predictor and may result in misleading conclusions [39, 40]. Indeed, a systematic review was unable to draw any conclusions regarding the predictive capacity of resting HRV for cardiorespiratory fitness in children and adolescents. However, the authors highlight important methodological and statistical issues that may have contributed to the lack of clarity regarding this outcome [41].

In obese adolescents, weak correlations were found between short-term resting HRV indices (SDNN, SD1, and SD2) and peak V̇O₂, and HRV did not emerge as a significant predictor in regression analysis [42]. However, in women with substance use disorders, both performance in the three-minute step test (3MST) and short-term resting HRV, assessed before and after the test, showed significant correlations with the peak V̇O₂ obtained during the cardiopulmonary exercise test (CPET) on a cycle ergometer, being independent predictors of this outcome. Among the variables analyzed, SDNN (ms) post-3MST stood out, explaining approximately 22% of the variance in peak V̇O₂ during the CPET in the entire sample [43]. However, when the sample was divided into two groups (Group 1: methamphetamine users with less than 10 years of experience; Group 2: methamphetamine users with 10 years or more of experience), SDNN post-3MST showed no significant estimated effect on peak V̇O₂ in those with longer methamphetamine use. It is important to note that this subgroup analysis involved a very small sample (*n* = 14), which may have influenced the absence of the observed effect. The small sample size could limit the statistical power of the analysis, making it more difficult to detect significant differences or effects, even if they exist in the general population. Furthermore, small samples may increase the risk of selection bias and random variability, which could affect the robustness and generalizability of the results [43].

Physiologically, the relationship between HRV and V̇O₂ may reflect integrated autonomic and cardiovascular responses to stress and exertion. In our study, SD2 associated with age, sex, and height demonstrated the strongest predictor of V̇O₂, reinforcing their potential utility as non-invasive markers of functional capacity. Furthermore, few studies implement cross-validation or report prediction intervals—steps essential for assessing clinical applicability. While our findings highlight the predictive value of specific HRV components, further research with improved statistical rigor is required before these variables can be established as reliable clinical predictors.

## Clinical impact

Resting HRV in individuals with mild post-COVID is significantly associated with and predictive of V̇O₂ during the 6MST. This finding supports HRV as a practical, low-cost and non-invasive surrogate marker of functional capacity. Given its prognostic relevance beyond cardiac autonomic assessment, HRV may enhance clinical decision-making, particularly in outpatient or resource-limited settings where exercise testing is impractical. Incorporating resting HRV into clinical evaluation may aid in early risk stratification and guide rehabilitation strategies in post-COVID care.

## Limitations and recommendations

This study has some limitations that may limit the generalizability of the results. First, while the use of a portable heart rate monitor offers practicality in clinical and field settings, it may be subject to signal variability and measurement error. Second, the predictive equation developed in this study has not undergone external validation, as it is an essential step to ensure model robustness and applicability in broader populations. Third, the cross-sectional design precludes causal inference and limits the ability to determine temporal relationships. Given that HRV is influenced by multiple factors, future studies should continue to account for potential confounders beyond those already included in our model, considering variables like physical activity level, medication use, and lifestyle factors. Larger, more representative samples and external validation are needed to improve the generalizability of findings. Additionally, standardizing HRV measurement protocols, including the duration of recordings (short- or long-term), controlled breathing, standardized body position, and exercise testing procedures, is recommended to reduce methodological heterogeneity commonly observed in the literature.

## Conclusion

Short-term resting HRV may serve as a predictor of V̇O₂ (mL·kg^−1^·min^−1^) during submaximal exercise in individuals recovering from mild COVID-19 without hospitalization. This study highlights the potential clinical utility of HRV in evaluating cardiorespiratory fitness in post-COVID-19 care. Beyond its established role in assessing autonomic function, HRV may serve as an accessible, non-invasive marker of functional capacity. Among the HRV parameters analyzed, SD2 (ms) emerged as the most relevant predictor of V̇O₂, even after adjusting for confounding variables such as age, sex, and body weight. Future studies should incorporate HRV into multivariate models alongside clinically accessible outcomes to enhance the understanding of V̇O₂ variability in longitudinal studies of this population.

## Methodology

### Study design

The present cross‑sectional investigation adheres to the STROBE statement for observational research [44]. Data collection was carried out at the Federal University of São Carlos (São Carlos, SP, Brazil) and its affiliated University Hospital (HU‑UFSCAR/EBSERH). Ethical approval was granted by the institution’s Research Ethics Committee (Approval No. 5 499 064), and all procedures conformed to the Declaration of Helsinki. Prior to participation, volunteers were fully briefed on the study’s aims and protocol and signed a written informed consent document.

### Participants

Men and women, ages 18 years or older, with COVID-19 infection (diagnosed by RT-PCR for SARS-CoV-2), occurring up to 6 weeks before the first visit, were recruited for this study. In order to define mild COVID-19 symptoms, we used the National Institutes of Health (NIH) COVID-19 treatment guideline that was adopted [45]: presence of signs and symptoms of the disease, such as fever, cough, sore throat, malaise, pain, headache, muscle pain, nausea, vomiting, diarrhea, loss of taste and smell, peripheral oxygen saturation (≥ 95%), and without shortness of breath and abnormal chest imaging.

Participants were excluded if they had been diagnosed with moderate [who presented to the hospital setting with clinical symptoms or radiologic evidence of lower respiratory tract disease and had an oxygen saturation (SpO₂) ≥ 94% on room air, but did not require hospitalization] to severe COVID-19, who were hospitalized [SpO_2_ less than 94% on room air, a ratio of partial pressure of arterial oxygen to fraction of inspired oxygen (PaO_2_/FiO_2_) of less than 300, tachypnea with a respiratory frequency of greater than 30 breaths/min, or lung infiltrates that are greater than 50% of total lung volume, or acute respiratory failure], experienced a myocardial infarction, received a pacemaker or metal implant, had a history of heart disease, unstable angina, uncontrolled hypertension or diabetes, chronic obstructive pulmonary disease or other respiratory diseases, neoplasms, cognitive impairment, reported illicit drug use, or were pregnant.

### Pulmonary function

A comprehensive lung function evaluation was completed with a whole‑body plethysmograph (Masterscreen Body, Mijnhardt/Jäger, Würzburg, Germany). Following ATS/ERS standards [46, 47], a trained examiner performed spirometry. The primary variables recorded were forced vital capacity in liters and percentage (FVC, L and %), forced expiratory volume in one second (FEV₁, L and %), and the FEV₁/FVC ratio. These percentages represent the ratio between the observed values and the predicted normal values. The reference values were established using prediction equations specific to the Brazilian population, taking into account age (years), sex, weight (kg), and height (cm) [48, 49].

### Standardized procedures for HRV collection and analysis: environmental control and experimental conditions

To minimize the influence of circadian rhythm fluctuations, all assessments were performed in the morning. Environmental and behavioral factors such as sleep and room lighting conditions were strictly standardized. Participants were instructed to follow a consistent sleep routine during the 24 h preceding the evaluation. Dietary intake was controlled, with a fasting period of at least 2 h prior to the assessment. Lighting in the evaluation room was kept constant to avoid exposure to intense light, which could interfere with circadian regulation. Additionally, participants were advised to abstain from consuming stimulants and from engaging in intense physical activity both on the day before and the day of the assessment. During data acquisition, participants remained in a supine position under controlled environmental conditions (temperature between 22 and 24 °C and relative humidity between 50 and 60%). Subjects were instructed to remain calm, breathe spontaneously, avoid talking or moving, and not to fall asleep. Prior to the recording, a 10-min rest period was observed to ensure heart rate stabilization. HRV data were collected over 10–15 min using the Polar H10 heart rate monitor (Polar Electro Oy Inc., Kempele, Finland) with a sampling rate of 1000 Hz. From this recording, a 5-min segment with the greatest signal stationarity was visually identified and selected for analysis. The HRV variables were extracted from linear analysis (time and frequency domains), and nonlinear analysis [7, 10, 11, 20, 50].

### HRV signal processing and segment selection

We used the Kubios HRV Standard software (version 3.5.0, University of Eastern Finland, https://www.kubios.com/) to process the heart rate variability (HRV) signals. The identified artifacts were removed manually or using the “very low” filter available in the software. This filter identifies RR intervals that deviate from the local average interval by more than 0.45 s and corrects them using cubic spline interpolation. The selection of this filter is supported by previous studies showing that it effectively corrects artifacts in middle-aged and adult populations without introducing significant distortions in HRV parameters [51–53]. The R–R interval series was detrended using the smoothing method, with a lambda value set to 500 and cubic interpolation at the default rate of 4 Hz. We then performed a detailed visual inspection of the recording and selected the segment with the greatest signal stationarity within a 5-min time window. The criteria for this selection were: (1) absence of outlier R–R intervals; (2) consistency of R–R intervals; and (3) approximately normal distribution in the R–R interval and heart rate graphs [11, 54].

### HRV parameters: time-domain, frequency-domain, and nonlinear metrics

#### Time-domain

The following variables were analyzed: the average R–R interval duration (mean RR) in ms, the standard deviation of all normal N–N intervals (SDNN) in milliseconds (ms), the mean heart rate (Mean HR) in beats per minute (bpm), the square root of successive mean squared differences of RR (RMSSD) in ms, the integral of the density of the RR interval histogram divided by its height (RR Tri), and the baseline width of a histogram displaying NN intervals (TINN) [10, 20, 55].

#### Frequency-domain

The frequency domain variables were determined through spectral analysis using the fast Fourier transform (FFT). The results of the frequency domain parameters were expressed as high frequency (HF: 0.15–0.40 Hz) and low frequency (LF: 0.04–0.15 Hz) components, presented in normalized units (n.u.) [10, 20, 55].

#### Nonlinear analysis

Nonlinear heart rate variability (HRV) analysis was conducted to extract the standard deviation perpendicular to the line of identity (SD1) and the standard deviation along the line of identity (SD2). Additionally, approximate entropy (ApEn) and sample entropy (SampEn) were assessed, along with detrended fluctuation analysis (DFA), which characterizes short-term (DFAα1) and long-term (DFAα2) HRV dynamics [10, 20, 55].

### Six-minute step test (6MST) protocol

**Fig. 1:**
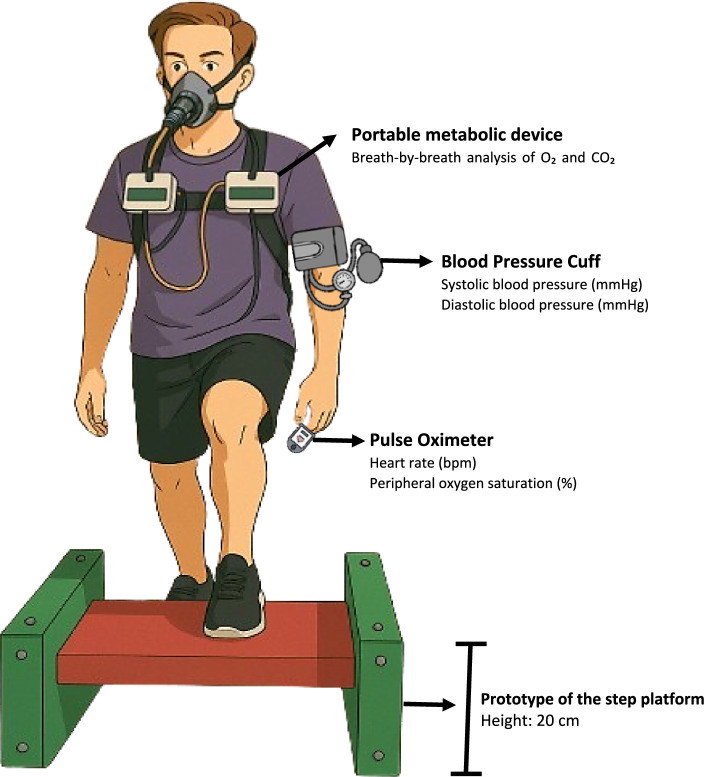
Schematic illustration of the 6MST. 6MST, six-minute step test; O2, oxygen; CO2, carbon dioxide; mmHg, millimeter of mercury; bpm, beats per minute; %, percentage; cm, centimeter. The illustration was developed by artificial intelligence based on a photograph owned by the authors. Sphygmomanometer and pulse oximeter used in the image, provided by Flaticon Credit: Icons created by Freepik–Flaticon

The 6MST was conducted according to the standard protocol outlined by the American Thoracic Society (ATS) for the six-minute walk test, adapted to the step format, since no specific guidelines have been published for the 6MST [56]. Considering that both tests assess functional capacity in a submaximal exercise context, the same safety and termination criteria described by the ATS were adopted, as well as the same standardized verbal encouragements provided at each minute. This included 4 min of rest (2 min seated followed by 2 min standing), 6 min of climbing up and down a 20‑cm step, and 6 min of seated recovery. Vital signs were measured in the initial seated rest and immediately at test completion: heart rate in beats per minute (bpm), peripheral oxygen saturation (SpO₂ %), systolic and diastolic blood pressure in millimeters of mercury (SBP and DBP, mmHg). Perceived dyspnea and leg fatigue were rated with the Borg 0–10 scale. Participants were advised that they could reduce their pace, pause, or rest if necessary, but should restart the test as soon as they felt able. Verbal encouragement was given each minute. Figure 1 presents a schematic illustration of the 6MST, including metabolic and ventilatory variables collection and vital signs monitoring.

Specific criteria were established to stop the test to ensure participant safety: SpO_2_ ≤ 87%, SBP > 220 mmHg, DBP > 110 mmHg, or the presence of symptoms such as dizziness, vertigo, or nausea [56]. Maximum heart rate (HRmax) was calculated using the formula: HRmax = 208 − (0.7 × age) [57]. Recovery heart rate was assessed at the first, third, and sixth minutes post-test, relative to the peak heart rate. The predictive equation developed by Albuquerque [58] was used to estimate the predicted percentage of the 6MST.

### Cardiorespiratory and metabolic responses

To assess the cardiorespiratory and metabolic responses, a portable telemetric gas analysis system (Oxycon Mobile Mijnhardt/Jager, Würzburg, Germany) was used, which performs continuous breath-by-breath analysis of expired gases during the test. Prior to the test, the CO_2_ and O_2_ analyzers were calibrated with a standard gas mixture (5% CO_2_, 12% O_2_, and nitrogen) and a reference gas, adjusted to room air under standard temperature and pressure dry (STPD) conditions. The pneumotachograph calibration was performed using a known flow generator, ensuring the accuracy of respiratory measurements. The following outcomes were collected for subsequent analysis: V̇O_2_ (mL·min^−1^), corrected for body weight (mL·kg^−1^·min^−1^), V̇CO_2_ (mL·min^−1^), minute ventilation (V̇_E_, L/min), respiratory rate (RR, breaths per minute), and the respiratory exchange ratio (RER) [59]. These parameters were calculated as the average of the last 20 s at the peak of the test.

### Statistical analysis

Results are reported as mean ± standard deviation (SD), median (interquartile range, 25–75%), or absolute count and percentage. Data normality was evaluated with the Shapiro–Wilk test  [60]. Because the dependent variable [V̇O₂ (mL·kg^−1^·min^−1^)] showed a non‑normal distribution, Spearman correlations were applied to quantify its association with each HRV parameter. The magnitude of the Spearman correlation coefficients was interpreted as follows: values from 0.00 to 0.10 indicate a negligible correlation, 0.10–0.39 represent a weak correlation, 0.40–0.69 suggest a moderate correlation, 0.70–0.89 indicate a strong correlation, and values between 0.90 and 1.00 reflect a very strong correlation [61]. The HRV variable showing the strongest correlation with V̇O₂ (mL·kg^−1^·min^−1^) was then entered into the multiple linear regression model, controlling for potential confounding variables, to estimate the strength and direction of its association. Only the most representative HRV variable (SD2) was included in the model to avoid overfitting and preserve the statistical power of the analysis, given the limited sample size (*n* = 45).

The final multiple regression model was then adjusted by enter method to improve the explanation of variance (adjusted R^2^). The covariates included in the regression analysis constituted a broad spectrum of factors associated with V̇O₂ (mL·kg^−1^·min^−1^), like age, weight, and sex [62, 63]. A *p* < 0.05 was adopted to demonstrate a statistical difference.

The homoscedasticity of the residuals was assessed by means of a scatter plot of the unstandardized residuals in relation to the fitted values. This assessment was performed to verify the assumption that the variance of the residuals is constant across the fitted values and to ensure the homoscedasticity assumption required for regression analysis [64]. We used the Durbin–Watson test to check for the presence of autocorrelation in the residuals in our regression model [65], where values < 2 indicate positive autocorrelation, while values > 2 indicate negative autocorrelation [65]. Collinearity between independent variables was assessed using the variance inflation factor (VIF) and tolerance: VIF below 10 and tolerance close to 1 were considered acceptable to rule out the presence of collinearity [66]. We considered a number of 10 participants for each independent variable added to the final multiple regression model [64, 67]. To verify the normality of the residuals of the regression model, the Shapiro–Wilk test for standardized residuals was used and a *p*-value > 0.05 was used to accept the hypothesis that the residuals are normally distributed.

All analyses were performed using GraphPad Software, Inc. (2019). GraphPad Prism (version 8.0.1). San Diego, CA (https://www.graphpad.com) .

## Data Availability

The datasets used and/or analyzed during the current study are available from the corresponding author on reasonable request.
